# Latinx: Sí, Se Puede? A Reflection on the Terms Past, Present, and Future

**DOI:** 10.1007/s11524-022-00690-y

**Published:** 2022-10-19

**Authors:** S. Raquel Ramos, Carmen J. Portillo, Christine Rodriguez, Jose I. Gutierrez

**Affiliations:** 1 Yale School of Nursing, 400 West Campus Drive, Orange, CT 06477 USA; 2 Department of Social and Behavioral Sciences, Yale School of Public Health, 60 College St., CT 06520 New Haven, USA; 3 Center for Interdisciplinary Research On AIDS, 135 College Street, Suite 200, New Haven, CT 06510 USA; 4 National Clinician Scholars Program, University of California, San Francisco, 3333 California St., CA 94118 San Francisco, USA

## Introduction

Latinos are the fastest growing ethnic minority segment of the population in the USA, and by the year 2060, it has been estimated that this population will reach 111.22 million [1]. In 2019, it was estimated that 60.5 million persons in the USA identified as having Latino/Hispanic ethnicity [1]. Broadly, Latino and Hispanic refer to persons with origins or heritage from the Caribbean (Cuba, Puerto Rico, etc.), Mexico, Spain, and Central and/or South America, regardless of race, and the terms are used interchangeably [2]. The purpose of this editorial is to describe the historic and current landscape of the term Latinx and provide future directions for greater awareness and inclusivity of the term.

A 2020 Pew Research Center report found that only 23% of US adults who self-identify as Latino have heard of the term Latinx and only 3% accept using the term [3, 4]. In 2021, a Gallup Poll suggested that there was no preferred ethnic subgroup label for Hispanic, although close to 25% reported preferring the term Hispanic and few reported preferring the term Latinx [5]. There have been multiple conflicting posts on social media about the term from persons of Hispanic heritage. Most recently, a LGBTQ congressional representative commented that they have not heard anyone local in the New York City/South Bronx area, which has one of the largest Hispanic concentrations in the USA, use the term [6]. Nonetheless, the receptivity toward the term continues to evolve as debates consider topics of gender and self-identify within Latino populations [7–9].

The term Latinx is not meant to replace Latina/o, but rather peacefully reflect inclusivity. The Spanish language is gender based and words in Spanish, by design, designate persons as either male or female, excluding persons who identify somewhere in between gender spectrums. It was first thought to originate within queer communities in 2004. However, by 2015, it prolifically appeared within social media and research outlets. Thus, “Latinx” has been operationalized as a non-binary term to circumvent the default use of masculine terminology that might not accurately represent one’s gender identity [10, 11].

In the USA, less than 5% of the Latino population use the non-binary term, Latinx [4]. To this point, Salinas conceptualized the term Latinx with *voces perdidas* and *voces de poder* [12]. Such a term not only brings voices to those individuals whose intersectionality may have been erased, but it also provides them with power [12]. As previously mentioned, the term Latinx is meant as an all-inclusive term, an extension of our vocabulary [13]. It is not intended to erase identities, but rather to bring them forth so that everyone has a voice that is heard. This applies in all areas of life and learning. According to Torres, there are various iterations of terminology circulating—Latinx, Latino, Latina, Latina/o, Latin@, Latin, Latin American, and Hispanic [11, 14]. Torres encourages writers to use any of the terms, but to be consistent throughout their writing, as well as provide a definition in a footnote for the readers that might not be familiar with the terminology in order to avoid unnecessary confusion regarding to the word of choice, in a manner that is both respectful and clear to the audience [14].

Within scholarly literature, Latinx was first traced back to a Puerto Rican publication in psychology aimed to challenge the current gender binary that exists within the Spanish language [13]. Early operationalization of the term by students was first documented when they changed their student group name from Chicano Caucus to Chicanx Caucus to include all gender identities/expressions [11]. However, some believe that this utilization has occurred without respect to acknowledgment of the various intersections (i.e., gender, gender identity/gender expressions, languages, folklore, cultures, histories, bodies, etc.) and memberships that our community holds. Salinas suggested that various arguments exist with regards to the term Latinx and its roots in Indigenous languages, and has been appropriated by dominant cultures [15]. However, despite the many connections that have been attempted to make between the term Latinx and Indigenous communities, there is no evidence that Latinx is related to any Indigenous communities outside of Mexico [16].

The widespread adoption of the term has since met resistance, with some considering the term as a form of linguistic imperialism or “Anglicizing” of the Spanish language. Opponents have further criticized “Latinx” as an example of a forced use of a term within a language and culture that had little input in its conceptualization, as well as its complicated use for its pronunciation within the spoken dialect. It has been seen as another way to further ignore the nuance and cultural differences of a diverse ethnic population in order to neatly classify these populations underneath one term [8].

The term Latinx allows others to ask questions concerning use of the term. According to Milian [17] and Torres [14], “the term *Latinx*—rather than Latino, Latina, Latina/o, Latin@, Latin, or Latin American—allows people to ask questions about the various intersections members of our community hold” [15]. This has, in turn, caused some discomfort in such areas, as Latinx represents something outside of societal norms. Latina/o have existed for a historical length of time and with this comes many strong beliefs that a binary approach to the term is the best and/or only approach that should be considered. Differences (contrasts) within culture, language, and access to education inhibit the consensus toward the implementation of the term Latinx and its use. As Salinas previously used, *voces perdidas* and *voces de poder*, he also stated that after conducting a study about the term Latinx and its use, “a majority of the participants perceived higher education as a privileged space where they only used the term *Latinx* to be inclusive [12]. Once they returned to their communities, they did not use the term, as they did not want to be the *voces de poder* overshadowing the *voces perdidas*” [15]. Overall, it can be said that the term Latinx can mean a plethora of things and represent many backgrounds, or nothing at all; it is all connected on how the individual uses the term that really matters [15, 18, 19].

## Conclusion

In the end, the history, intentions, and operationality of the term Latinx are varied and complex, and envisioning a unified solution that comprehensively addresses the critiques of the term “Latinx “appears equally complicated. More certain, however, is the value in the examination of novel solutions that replace gendered language within the healthcare setting, and the positive implications that gender-neutral communication can have on mitigating clinical bias, education reform, and improvement of patient interactions. In the interim, consideration may be given to allow space for authors to use terminology (e.g., Latinx, Latina/o/e, Latin@) that they feel best represents their identity and community, with a foundation of understanding that the reasons for preferred terminology may be as diverse as the cultures that the term attempts to embody. As society evolves, so should the ways in language is used and also the terms the people use to best describe themselves.

## References

[1] Census US. Hispanic population to reach 111 million by 2060. In: The United States Census Bureau. 2018. https://www.census.gov/library/visualizations/2018/comm/hispanic-projected-pop.html. Accessed 4 June 2022.

[2] Census US. About the Hispanic population and its origin. In: The United States Census Bureau. https://www.census.gov/library/visualizations/2018/comm/hispanic-projected-pop.html. Accessed 4 June 2022.

[3] Noe-Bustamante L, Mora L, Lopez M. Views on Latinx as a pan-ethnic term for US Hispanics. In: Pew Research Center. https://www.pewresearch.org/hispanic/2020/08/11/views-on-latinx-as-a-pan-ethnic-term-for-u-s-hispanics/. Accessed 7 May 2022.

[4] Noe-Bustamante L, Mora L, Lopez MH. About one-in-four US Hispanics have heard of Latinx, but just 3% use it. Pew Research Center. 2020;11. https://www.pewresearch.org/hispanic/2020/08/11/about-one-in-four-u-s-hispanics-have-heard-of-latinx-but-just-3-use-it/. Accessed 4 June 2022.

[5] Gallup. No preferred racial term among most Black, Hispanic Adults. 2021. https://news.gallup.com/poll/353000/no-preferred-racial-term-among-black-hispanic-adults.aspx. Accessed 4 June 2022.

[6] Patteson C. *South Bronx Rep. Torres knocks Yankees over 'Latinx' tweet: 'Never heard anyone use' it*. New York Post. May 30, 2022, 2022. https://nypost.com/2022/05/30/south-bronx-rep-torres-knocks-yankees-over-use-of-latinx/.

[7] Brammer JP. Digging into the messy history of “Latinx” helped me embrace my complex identity. Mother Jones. 2020. https://www.motherjones.com/media/2019/06/digging-into-the-messy-history-of-latinx-helped-me-embrace-my-complex-identity/. Accessed 1 July 2022

[8] Guerra G, Orbea G. The argument against the use of the term “Latinx.”. The Phoenix. 2015;19. https://swarthmorephoenix.com/2015/11/19/the-argument-against-the-use-of-the-term-latinx/. Accessed 2 July 2022.

[9] Alamo HL. *The X-ing of language: The case AGAINST ‘Latinx*.’ Latino Rebels. 2015. https://www.latinorebels.com/2015/12/12/the-x-ing-of-language-the-case-against-latinix/.

[10] Simón Y. Latino, Hispanic, Latinx, Chicano: The History Behind the Terms. History com. 2020. https://www.historycom/news/hispanic-latino-latinx-chicano-background. Accessed 29 May 2022.

[11] Armus T. *Student groups shift toward use of Latinx to include all gender identities*. Columbia Daily Spectator. 2015. p. 8. https://www.columbiaspectator.com/news/2015/10/07/latino-latinx/.

[12] Salinas C (2017). Transforming academia and theorizing spaces for Latinx in higher education: voces perdidas and voces de poder. Int J Qual Stud Educ.

[13] Logue J. Latina/o/x. 2015; https://www.insidehighered.com/news/2015/12/08/students-adopt-gender-nonspecific-term-latinx-be-more-inclusive. Accessed 5 Mar 2022.

[14] Torres L. Latinx?. Lat Stud 16, 283–285 (2018). 10.1057/s41276-018-0142-y.

[15] Salinas C (2020). The complexity of the “x” in Latinx: how Latinx/a/o students relate to, identify with, and understand the term Latinx. J Hisp High Educ.

[16] Santos CE (2017). The history, struggles, and potential of the term Latinx. Latina/o Psychol Today.

[17] Milian C. *Extremely Latin, XOXO: Notes on LatinX*. Cultural Dynamics. 2017;29(3):121–140. 10.1177/0921374017727850.

[18] DeGuzmán M (2017). Latinx:¡ Estamos aquí!, or being “Latinx” at UNC-Chapel Hill. Cult Dyn.

[19] Galvan R (2017). EE/UU: exquisite expression/unsettling utterance. Cult Dyn.

